# 

**Published:** 1833-01-01

**Authors:** 


### CONTENTS

OF THE

### MEDICO-CHIRURGICAL REVIEW

### No. XXXV. JANUARY 1, 1833.

### REVIEWS.

I.

The Influence of Physical Agents on Life. By Dr. EDWARDS; translated by Dr. HODGKIN .. .. .. .. .. .. .. .. .. .. .. .. .. .. .. 1

II.

Medico-Chirurgical Transactions, Vol. XVII. .. .. .. .. .. .. .. .. .. .. 33

1. Mr. BRODIE on Abscess in the Interior of the Tibia .. .. .. .. .. .. 33
2. Experiments on the Use of Styptics in Hæmorrhage. By CÆSAR HAWKINS, Esq. .. .. .. .. .. .. .. .. .. .. .. .. .. .. .. .. 38
3. On the Phenomena and Appearances from partial Obstruction of the Cerebral Circulation. By Mr. HOWSHIP .. .. .. .. .. .. .. .. .. 44
4. Cases of Ovarian Disease, complicated with Pregnancy. By Mr. HEWLETT .. .. .. .. .. .. .. .. .. .. .. .. .. .. .. .. .. 46

III.

Recherches Physiologiques et Medicales sur les Causes et les Symptomes, et le Traitement de la Gravelle. Par F. MAGENDIE .. .. .. .. .. .. .. .. 49

IV.

A Treatise on the Epidemic Cholera. By F. CORBYN, Esq. .. .. .. .. .. .. 63

V.

A Theoretical and Practical Treatise on Ligature of Arteries. By Dr. MANEC .. 67

VI.

Researches on the Mechanism of the Human Voice. By F. BENNATI .. .. .. .. 79

VII.

M. SABATIER on the Laws of Revulsion.. .. .. .. .. .. .. .. .. .. .. .. .. 87

VIII.

New Theory of the Influence of Variety in Diet in Health and Disease. By Mr. CAMERON .. .. .. .. .. .. .. .. .. .. .. .. .. .. .. .. .. 94

IX.

Mr. BRANSBY COOPER's Lectures on Anatomy, Vol. IV. .. .. .. .. .. .. .. .. 97

X.

The Effects of Arts, Trades, and Professions, &c. on Health and Longevity. By Mr. THACKRAH .. .. .. .. .. .. .. .. .. .. .. .. .. .. .. .. .. 101

XI.

A Dictionary of Practical Medicine. By Dr. Copland. Part I. .. .. .. .. 111

XII.

Memoir on the Pearly Nautilus. By Richard Owen, Esq. .. .. .. .. .. 123

XIII.

Clinical Reports of the Surgical Practice of the Glasgow Royal Infirmary. By John Macfarlane, M.D... .. .. .. .. .. .. .. .. .. .. .. 126

XIV.

Dr. Hamett's Official Medical Reports on Cholera .. .. .. .. .. .. .. 136

XV.

Observations on the Medical Treatment of Insanity. By Dr. Seymour.. .. .. 139

### PERISCOPE.

1. Dr. Hancock on External Stimulants .. .. .. .. .. .. .. .. .. 145
2. Choleraphobia, dreadful Effects of .. .. .. .. .. .. .. .. .. 148
3. Medical Report from Dublin Fever Hospital.. .. .. .. .. .. .. 149
4. Dr. M'Cormac on Cholera .. .. .. .. .. .. .. .. .. .. 152
5. Influenza Epidemica .. .. .. .. .. .. .. .. .. .. .. 154
6. Clinical Observations on the Exhibition of large Doses of Opium. By Dr. Stokes .. .. .. .. .. .. .. .. .. .. .. .. 156
7. Clinical Report from St. Thomas's Hospital (from our own Reporter) .. .. 160
   1. Spurious Hydrocephalus .. .. .. .. .. .. .. .. .. .. 160
   2. Compound Fracture of Os Femoris .. .. .. .. .. .. .. .. 162
   3. Remarkable Cases of Dislocation .. .. .. .. .. .. .. .. 165
   4. Osteo Sarcoma .. .. .. .. .. .. .. .. .. .. .. .. 166
   5. Exostosis of unusual Magnitude .. .. .. .. .. .. .. .. 167
8. Counteraction used as a Means of Cure, with Remarks, &c. By Dr. Epps .. 170
9. M. Carus on the Evolution of the Chick.. .. .. .. .. .. .. .. 171
10. Gastro-enteritis simulating Cholera .. .. .. .. .. .. .. .. 173
11. Abscess in the Substance of the Heart .. .. .. .. .. .. .. .. 174
12. Erysipelas treated by Mercury .. .. .. .. .. .. .. .. .. 175
13. M. Broussais on large Doses of Antimony in Pneumonia .. .. .. .. .. 175
14. Diabetes Mellitus .. .. .. .. .. .. .. .. .. .. .. .. 175
15. Endermic Therapeutics .. .. .. .. .. .. .. .. .. .. .. 176
16. Method of dressing a Stump .. .. .. .. .. .. .. .. .. .. 176
17. Mania Pellagrina .. .. .. .. .. .. .. .. .. .. .. .. 176
18. The Bills of Mortality in Paris.. .. .. .. .. .. .. .. .. .. 181
19. Abscess of the Lung, communicating with the Surface .. .. .. .. .. 182
20. Spontaneous Gangrene .. .. .. .. .. .. .. .. .. .. .. 182
21. Curious Case of Poisoning .. .. .. .. .. .. .. .. .. .. 183
22. Various Modes of treating Cholera in Paris .. .. .. .. .. .. .. 184
23. Cholera Anecdotes .. .. .. .. .. .. .. .. .. .. .. .. 186
24. M. Sabatier on the premonitory Affections of Cholera.. .. .. .. .. .. 186
25. Fissure of the Anus .. .. .. .. .. .. .. .. .. .. .. .. 187

26. Curious Mode of applying Argentum Nitratum to the Eye.. .. .. .. .. 187
27. M. Dupuytren on the Contagion of Diseases.. .. .. .. .. .. .. .. .. 187
28. Rhinoplastic Operation .. .. .. .. .. .. .. .. .. .. .. .. .. .. .. 188
29. On the Changes which the Points of the Fingers undergo in Phthisis .. .. 189
30. Cases of Disease of the Kidneys .. .. .. .. .. .. .. .. .. .. .. .. 190
31. Dothinenterite .. .. .. .. .. .. .. .. .. .. .. .. .. .. .. .. .. 193
32. Laryngeal Fistula cured by Operation .. .. .. .. .. .. .. .. .. .. 194
33. M. Malapert on Syphilis .. .. .. .. .. .. .. .. .. .. .. .. .. .. 194
34. Cancer of the Tongue .. .. .. .. .. .. .. .. .. .. .. .. .. .. .. 194
35. Dr. Bryce on the Etiology of Cholera .. .. .. .. .. .. .. .. .. .. 195
Mr. Watt on the Etiology of Cholera .. .. .. .. .. .. .. .. .. .. .. 198
36. Dr. Lawrie on the proximate Cause of Cholera .. .. .. .. .. .. .. .. 199
37. Dr. Clendinning on the Effects of Cold.. .. .. .. .. .. .. .. .. .. 200
38. On the Employment of Tepid Baths, combined with Cold Affusions, in Cerebral Affections .. .. .. .. .. .. .. .. .. .. .. .. .. .. .. .. .. .. 205
39. Vaginal Cystocele .. .. .. .. .. .. .. .. .. .. .. .. .. .. .. .. 208
40. Case of Anencephalism .. .. .. .. .. .. .. .. .. .. .. .. .. .. .. 209
41. On Muscular Contraction and Animal Electricity .. .. .. .. .. .. .. 211
42. On the Phenomena to which the Terms Endosmosis and Exosmosis have been applied .. .. .. .. .. .. .. .. .. .. .. .. .. .. .. .. .. .. .. 213
43. Anatomy and Uses of the Lymphatics .. .. .. .. .. .. .. .. .. .. .. 214
44. Transmissibility of Diseases along an Electric Wire .. .. .. .. .. .. 215
45. Cholera in Scotland—Decision of the Dumfries Medical Meeting .. .. .. 216
46. Injury of the Penis, with Amputation .. .. .. .. .. .. .. .. .. .. 223
47. Removal of Calculus from Female Bladders, by Weiss's Instrument.. .. .. 224
48. Physic and Surgery? Professor Cooper .. .. .. .. .. .. .. .. .. .. 225
49. Clinical Instruction .. .. .. .. .. .. .. .. .. .. .. .. .. .. .. 226
50. Mr. Carmichael on Tracheotomy .. .. .. .. .. .. .. .. .. .. .. .. 227
51. Mercury in Chronic Affections of the Larynx .. .. .. .. .. .. .. .. 231
52. The Doctor deceived.. .. .. .. .. .. .. .. .. .. .. .. .. .. .. .. 231
53. The Actual Cautery in Vesico-vaginal Fistula. By Dr. Kennedy .. .. .. 232
54. Mr. Kerr on Glossitis.. .. .. .. .. .. .. .. .. .. .. .. .. .. .. 234
55. Enormous Tumour attached to the Ilium. By Mr. Bell .. .. .. .. .. .. 236
56. Mr. Cramptom on Inflammation of the Brain and its Membranes .. .. .. 237
57. Mr. Quain's Elements of Anatomy .. .. .. .. .. .. .. .. .. .. .. .. 242
58. What is the Contagion of Cholera? By Dr. Kennedy, of Ashby-de-la-Zouche 245
59. Ligature of the Subclavian Artery .. .. .. .. .. .. .. .. .. .. .. 248
60. Death after the Introduction of the Catheter .. .. .. .. .. .. .. .. 249
61. Dr. Wise on Wounds of the Intestines .. .. .. .. .. .. .. .. .. .. 250
62. Ligature of the Carotid for Hemiplegia and Epilepsy. By Mr. Preston.. .. 253
63. Surgical Anatomy of the Arteries .. .. .. .. .. .. .. .. .. .. .. .. 255
64. The Anatomy of the Horse .. .. .. .. .. .. .. .. .. .. .. .. .. .. 261
65. Excerpta Choleralogica .. .. .. .. .. .. .. .. .. .. .. .. .. .. .. 261
1. Dr. Haslewood and Mr. Mordey on Cholera .. .. .. .. .. .. .. .. 261
2. Dr. Keir on Cholera.. .. .. .. .. .. .. .. .. .. .. .. .. .. .. 262
3. Mr. Morrah on Sulphate of Copper in Cholera .. .. .. .. .. .. .. 263
4. Dr. Tweedie on Cholera.. .. .. .. .. .. .. .. .. .. .. .. .. .. 263
5. Mr. Steward on Cholera.. .. .. .. .. .. .. .. .. .. .. .. .. .. 264

6. Dr. Ayre on Cholera .. .. .. .. .. .. .. .. .. .. .. .. .. 264
7. Mr. Hickman on Cholera .. .. .. .. .. .. .. .. .. .. .. .. 264
8. Messrs Tweedie and Gaselee .. .. .. .. .. .. .. .. .. .. .. 265
9. Cholera in Dumfries .. .. .. .. .. .. .. .. .. .. .. .. .. 268
10. Choleraphobia in Ireland .. .. .. .. .. .. .. .. .. .. .. 268
11. Dr. Thompson on Cholera .. .. .. .. .. .. .. .. .. .. .. 269
12. New Proofs of the Veracity of Contagionists .. .. .. .. .. .. 271
13. Charcoal in Cholera .. .. .. .. .. .. .. .. .. .. .. .. .. 272
14. Dr. Williams and Dr. Baird .. .. .. .. .. .. .. .. .. .. .. 272
66. The Poisonous or Toad Fish of Van Dieman's Land .. .. .. .. .. .. .. 273
67. Hospital Report from St. George's Hospital .. .. .. .. .. .. .. .. 274
68. Weber's Anatomical Atlas .. .. .. .. .. .. .. .. .. .. .. .. 283
69. Dr. Hope's Work on Pathology .. .. .. .. .. .. .. .. .. .. .. 284
Bibliographical Record .. .. .. .. .. .. .. .. .. .. .. .. .. .. 286

